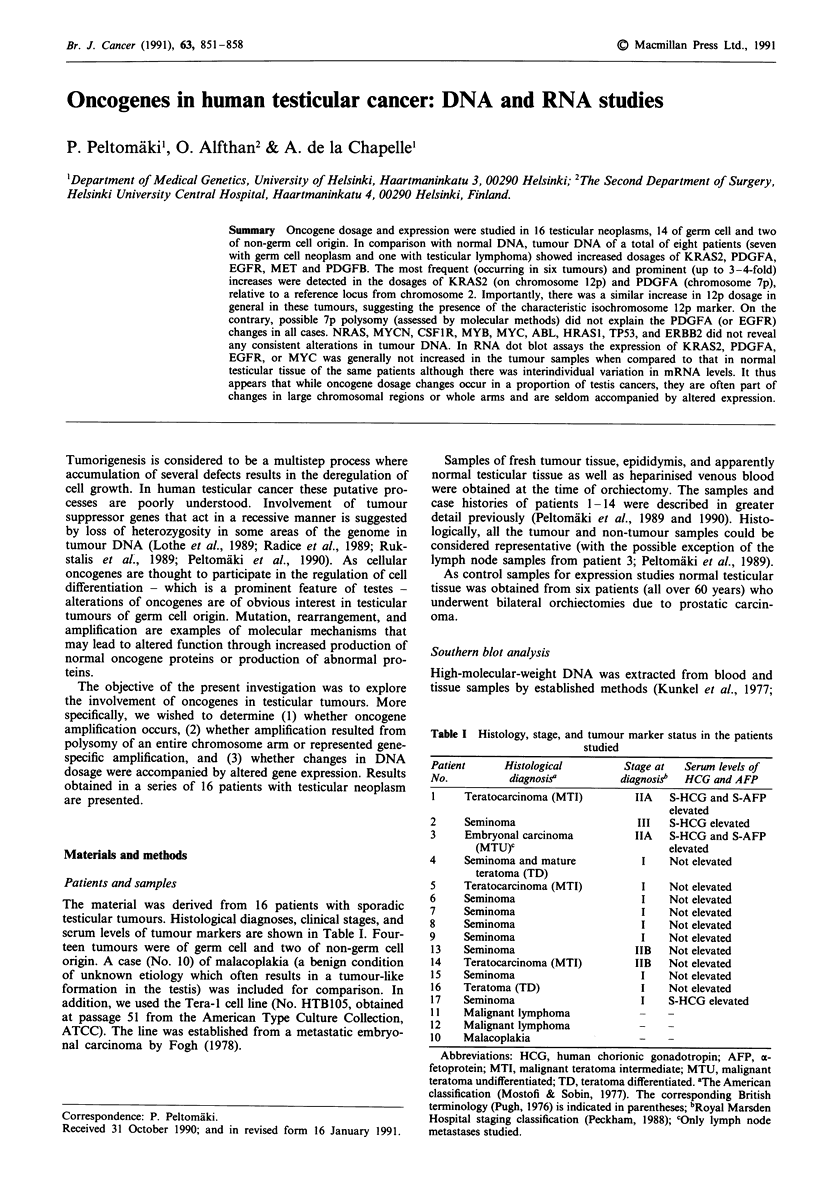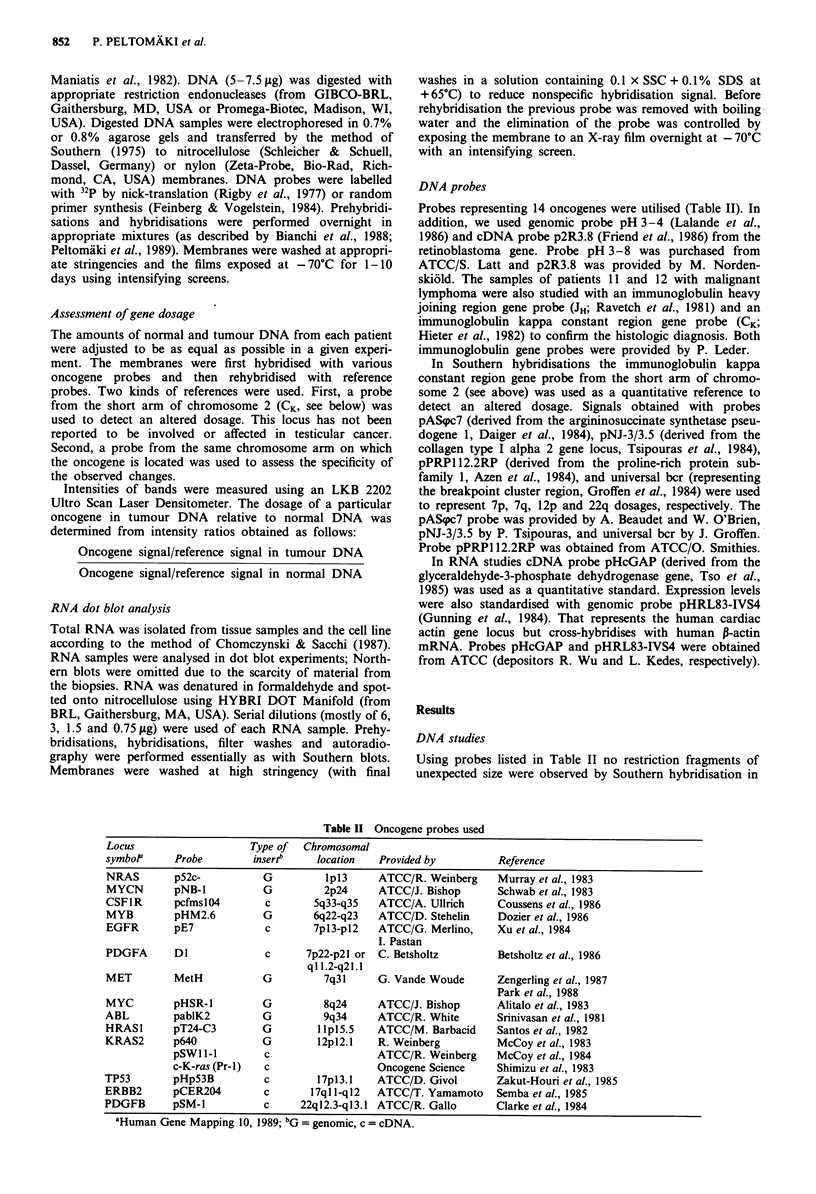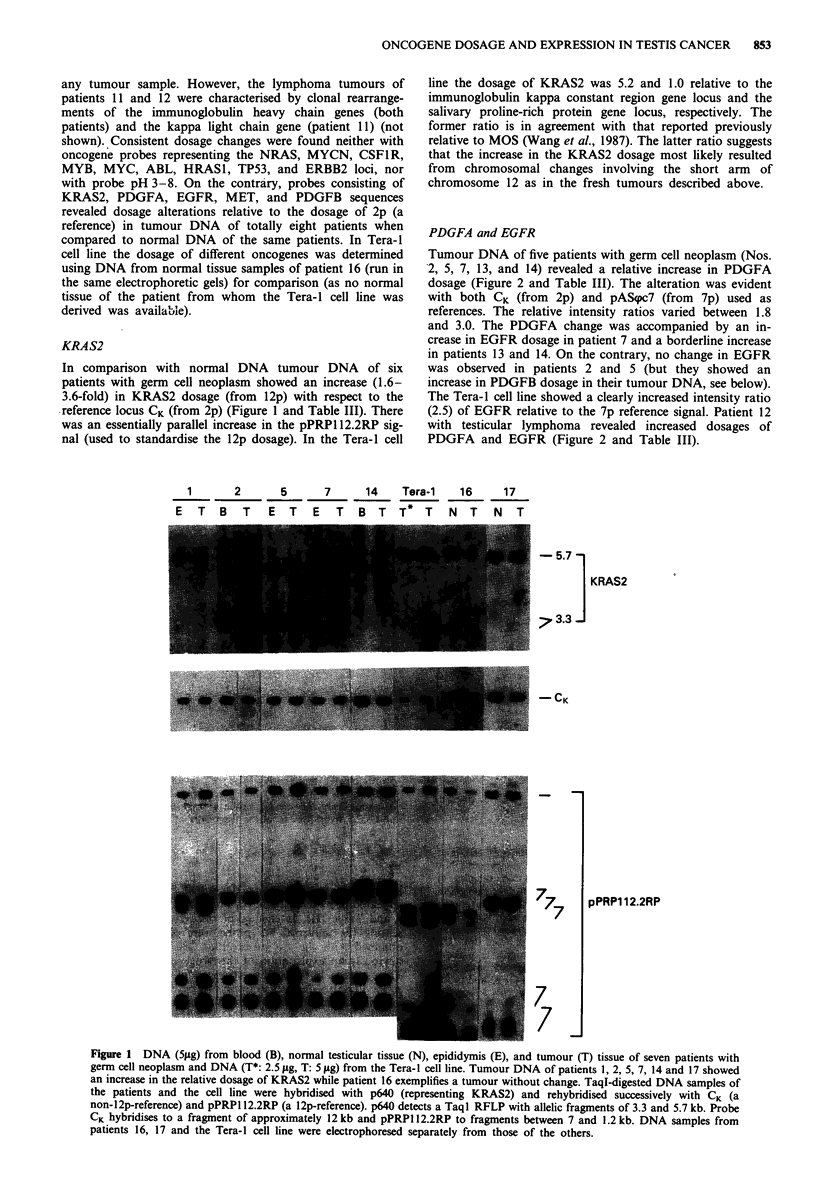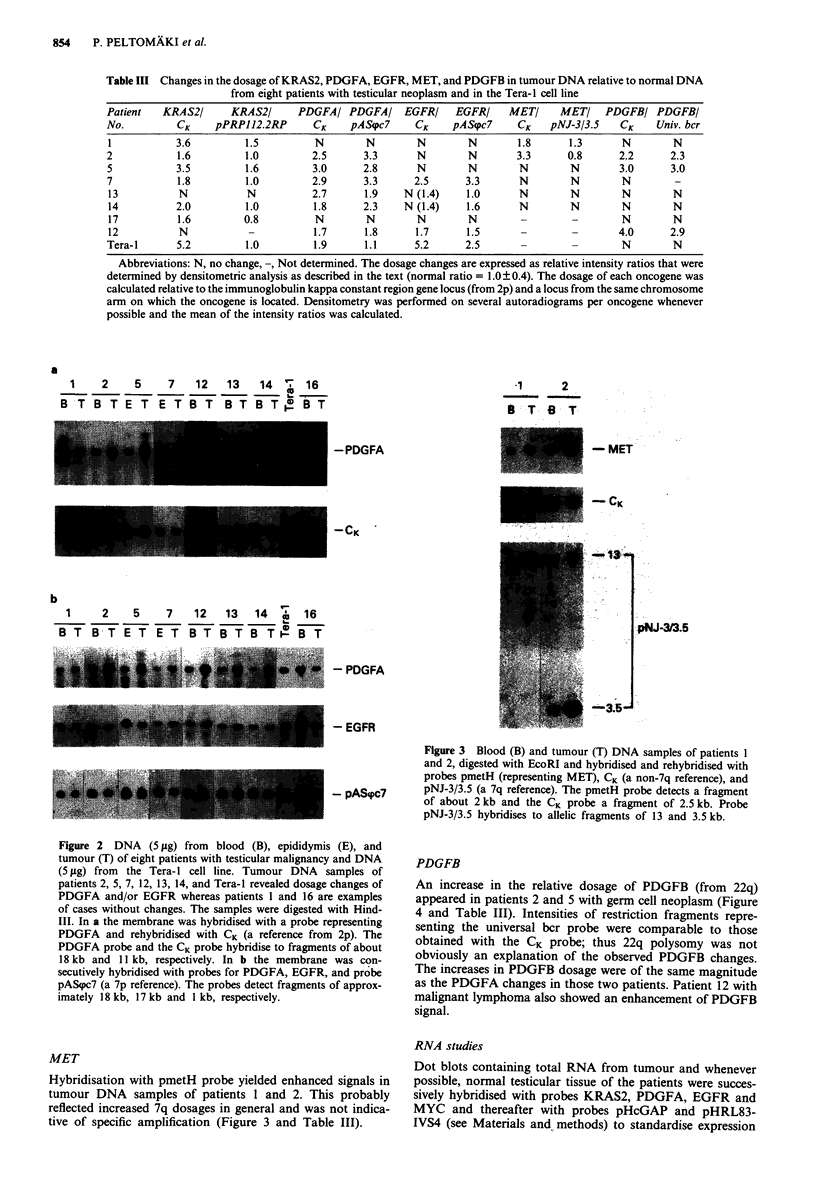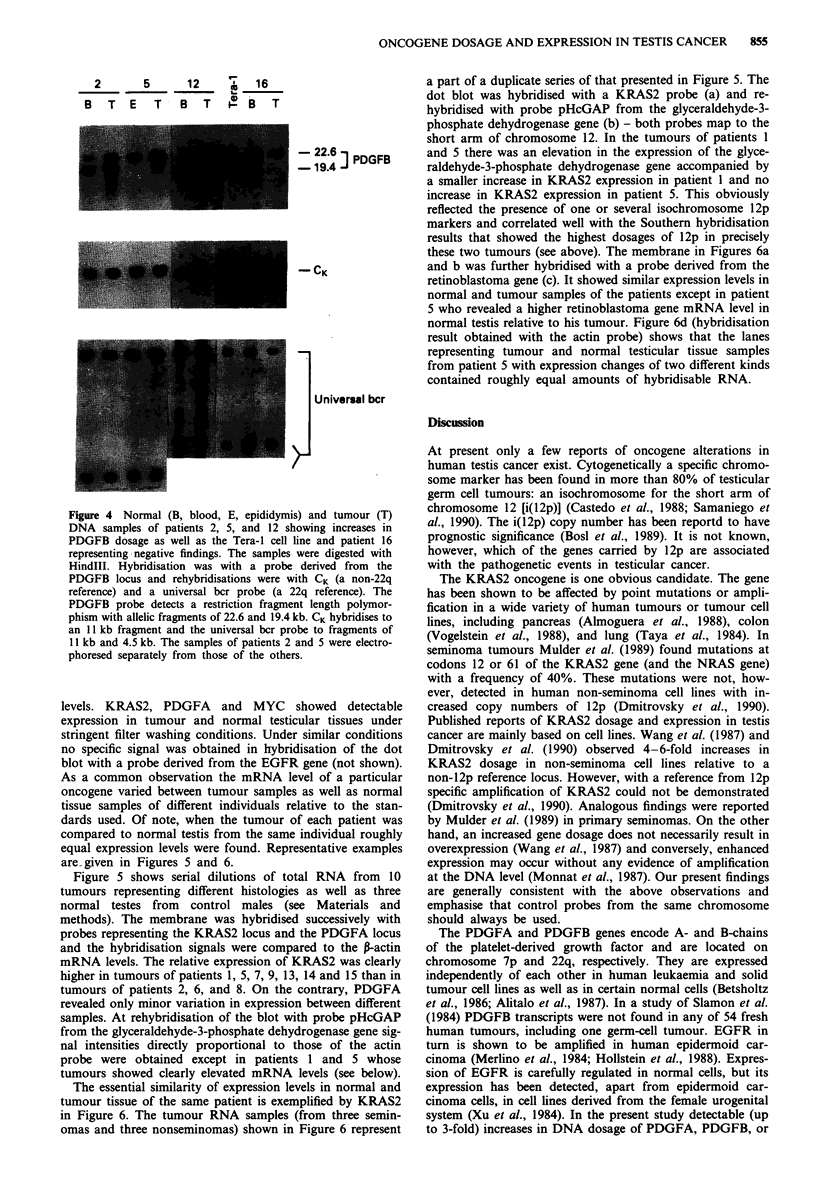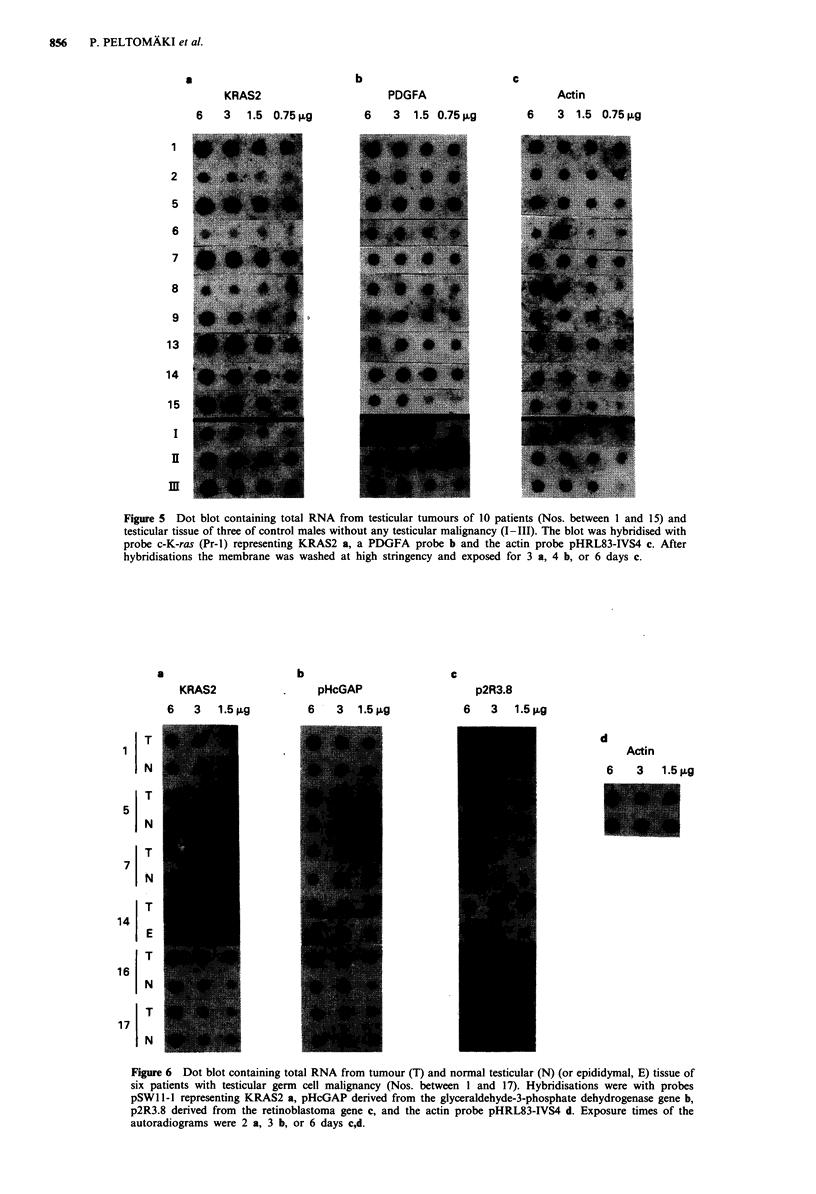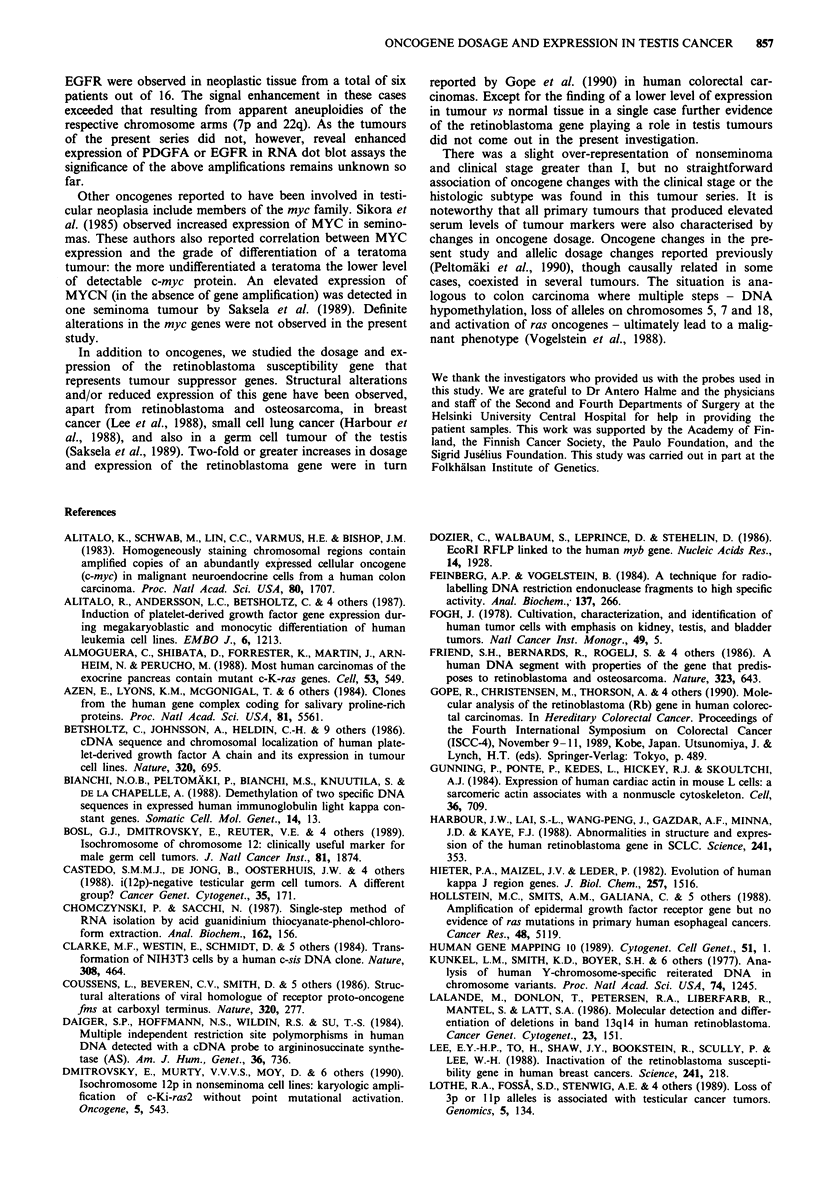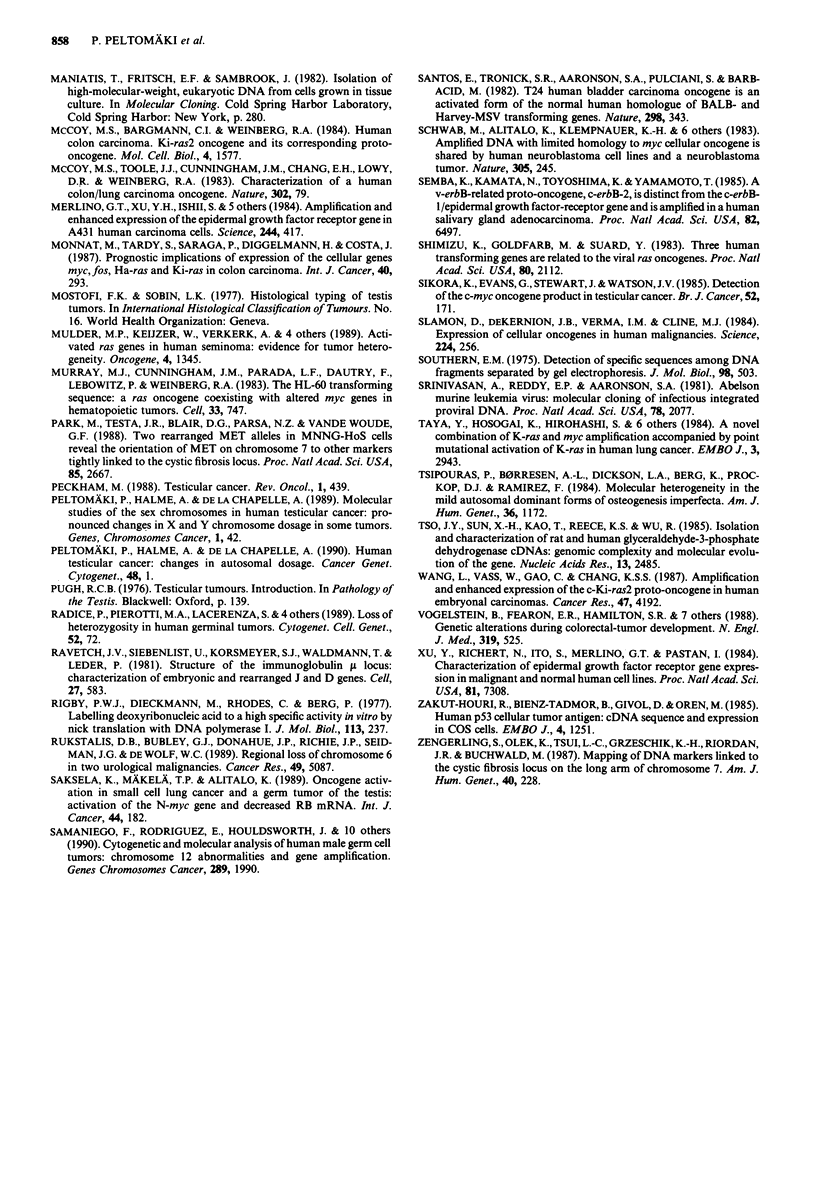# Oncogenes in human testicular cancer: DNA and RNA studies.

**DOI:** 10.1038/bjc.1991.189

**Published:** 1991-06

**Authors:** P. Peltomäki, O. Alfthan, A. de la Chapelle

**Affiliations:** Department of Medical Genetics, University of Helsinki, Finland.

## Abstract

**Images:**


Br. J. Cancer (1991), 63, 851-858 © Macmillan Press Ltd., 1991

### Oncogenes in human testicular cancer: DNA and RNA studies

P. Peltomäki[1], O. Alfthan[2] & A. de la Chapelle[1]

[1]*Department of Medical Genetics, University of Helsinki, Haartmaninkatu 3, 00290 Helsinki;* [2]*The Second Department of Surgery, Helsinki University Central Hospital, Haartmaninkatu 4, 00290 Helsinki, Finland.*

**Summary** Oncogene dosage and expression were studied in 16 testicular neoplasms, 14 of germ cell and two of non-germ cell origin. In comparison with normal DNA, tumour DNA of a total of eight patients (seven with germ cell neoplasm and one with testicular lymphoma) showed increased dosages of KRAS2, PDGFA, EGFR, MET and PDGFB. The most frequent (occurring in six tumours) and prominent (up to 3-4-fold) increases were detected in the dosages of KRAS2 (on chromosome 12p) and PDGFA (chromosome 7p), relative to a reference locus from chromosome 2. Importantly, there was a similar increase in 12p dosage in general in these tumours, suggesting the presence of the characteristic isochromosome 12p marker. On the contrary, possible 7p polysomy (assessed by molecular methods) did not explain the PDGFA (or EGFR) changes in all cases. NRAS, MYCN, CSF1R, MYB, MYC, ABL, HRAS1, TP53, and ERBB2 did not reveal any consistent alterations in tumour DNA. In RNA dot blot assays the expression of KRAS2, PDGFA, EGFR, or MYC was generally not increased in the tumour samples when compared to that in normal testicular tissue of the same patients although there was interindividual variation in mRNA levels. It thus appears that while oncogene dosage changes occur in a proportion of testis cancers, they are often part of changes in large chromosomal regions or whole arms and are seldom accompanied by altered expression.

Tumorigenesis is considered to be a multistep process where accumulation of several defects results in the deregulation of cell growth. In human testicular cancer these putative processes are poorly understood. Involvement of tumour suppressor genes that act in a recessive manner is suggested by loss of heterozygosity in some areas of the genome in tumour DNA (Lothe *et al.*, 1989; Radice *et al.*, 1989; Rukstalis *et al.*, 1989; Peltomäki *et al.*, 1990). As cellular oncogenes are thought to participate in the regulation of cell differentiation – which is a prominent feature of testes – alterations of oncogenes are of obvious interest in testicular tumours of germ cell origin. Mutation, rearrangement, and amplification are examples of molecular mechanisms that may lead to altered function through increased production of normal oncogene proteins or production of abnormal proteins.

The objective of the present investigation was to explore the involvement of oncogenes in testicular tumours. More specifically, we wished to determine (1) whether oncogene amplification occurs, (2) whether amplification resulted from polysomy of an entire chromosome arm or represented gene-specific amplification, and (3) whether changes in DNA dosage were accompanied by altered gene expression. Results obtained in a series of 16 patients with testicular neoplasm are presented.

### Materials and methods

### *Patients and samples*

The material was derived from 16 patients with sporadic testicular tumours. Histological diagnoses, clinical stages, and serum levels of tumour markers are shown in Table I. Fourteen tumours were of germ cell and two of non-germ cell origin. A case (No. 10) of malacoplakia (a benign condition of unknown etiology which often results in a tumour-like formation in the testis) was included for comparison. In addition, we used the Tera-1 cell line (No. HTB105, obtained at passage 51 from the American Type Culture Collection, ATCC). The line was established from a metastatic embryonal carcinoma by Fogh (1978).

Samples of fresh tumour tissue, epididymis, and apparently normal testicular tissue as well as heparinised venous blood were obtained at the time of orchiectomy. The samples and case histories of patients 1-14 were described in greater detail previously (Peltomäki *et al.*, 1989 and 1990). Histologically, all the tumour and non-tumour samples could be considered representative (with the possible exception of the lymph node samples from patient 3; Peltomäki *et al.*, 1989).

As control samples for expression studies normal testicular tissue was obtained from six patients (all over 60 years) who underwent bilateral orchiectomies due to prostatic carcinoma.

### *Southern blot analysis*

High-molecular-weight DNA was extracted from blood and tissue samples by established methods (Kunkel *et al.*, 1977;

**Table I** Histology, stage, and tumour marker status in the patients studied

| *Patient No.* | *Histological diagnosis*[a] | *Stage at diagnosis*[b] | *Serum levels of HCG and AFP* |
|---|---|---|---|
| 1 | Teratocarcinoma (MTI) | IIA | S-HCG and S-AFP elevated |
| 2 | Seminoma | III | S-HCG elevated |
| 3 | Embryonal carcinoma (MTU)[c] | IIA | S-HCG and S-AFP elevated |
| 4 | Seminoma and mature teratoma (TD) | I | Not elevated |
| 5 | Teratocarcinoma (MTI) | I | Not elevated |
| 6 | Seminoma | I | Not elevated |
| 7 | Seminoma | I | Not elevated |
| 8 | Seminoma | I | Not elevated |
| 9 | Seminoma | I | Not elevated |
| 13 | Seminoma | IIB | Not elevated |
| 14 | Teratocarcinoma (MTI) | IIB | Not elevated |
| 15 | Seminoma | I | Not elevated |
| 16 | Teratoma (TD) | I | Not elevated |
| 17 | Seminoma | I | S-HCG elevated |
| 11 | Malignant lymphoma | – | – |
| 12 | Malignant lymphoma | – | – |
| 10 | Malacoplakia | – | – |

Abbreviations: HCG, human chorionic gonadotropin; AFP, α-fetoprotein; MTI, malignant teratoma intermediate; MTU, malignant teratoma undifferentiated; TD, teratoma differentiated. [a]The American classification (Mostofi & Sobin, 1977). The corresponding British terminology (Pugh, 1976) is indicated in parentheses; [b]Royal Marsden Hospital staging classification (Peckham, 1988); [c]Only lymph node metastases studied.

Correspondence: P. Peltomäki.
Received 31 October 1990; and in revised form 16 January 1991.

Maniatis et al., 1982). DNA (5–7.5 μg) was digested with appropriate restriction endonucleases (from GIBCO-BRL, Gaithersburg, MD, USA or Promega-Biotec, Madison, WI, USA). Digested DNA samples were electrophoresed in 0.7% or 0.8% agarose gels and transferred by the method of Southern (1975) to nitrocellulose (Schleicher & Schuell, Dassel, Germany) or nylon (Zeta-Probe, Bio-Rad, Richmond, CA, USA) membranes. DNA probes were labelled with $^{32}P$ by nick-translation (Rigby et al., 1977) or random primer synthesis (Feinberg & Vogelstein, 1984). Prehybridisations and hybridisations were performed overnight in appropriate mixtures (as described by Bianchi et al., 1988; Peltomäki et al., 1989). Membranes were washed at appropriate stringencies and the films exposed at −70°C for 1–10 days using intensifying screens.

### Assessment of gene dosage

The amounts of normal and tumour DNA from each patient were adjusted to be as equal as possible in a given experiment. The membranes were first hybridised with various oncogene probes and then rehybridised with reference probes. Two kinds of references were used. First, a probe from the short arm of chromosome 2 ($C_K$, see below) was used to detect an altered dosage. This locus has not been reported to be involved or affected in testicular cancer. Second, a probe from the same chromosome arm on which the oncogene is located was used to assess the specificity of the observed changes.

Intensities of bands were measured using an LKB 2202 Ultro Scan Laser Densitometer. The dosage of a particular oncogene in tumour DNA relative to normal DNA was determined from intensity ratios obtained as follows:

$$\frac{\text{Oncogene signal/reference signal in tumour DNA}}{\text{Oncogene signal/reference signal in normal DNA}}$$

### RNA dot blot analysis

Total RNA was isolated from tissue samples and the cell line according to the method of Chomczynski & Sacchi (1987). RNA samples were analysed in dot blot experiments; Northern blots were omitted due to the scarcity of material from the biopsies. RNA was denatured in formaldehyde and spotted onto nitrocellulose using HYBRI DOT Manifold (from BRL, Gaithersburg, MA, USA). Serial dilutions (mostly of 6, 3, 1.5 and 0.75 μg) were used of each RNA sample. Prehybridisations, hybridisations, filter washes and autoradiography were performed essentially as with Southern blots. Membranes were washed at high stringency (with final washes in a solution containing 0.1 × SSC + 0.1% SDS at +65°C) to reduce nonspecific hybridisation signal. Before rehybridisation the previous probe was removed with boiling water and the elimination of the probe was controlled by exposing the membrane to an X-ray film overnight at −70°C with an intensifying screen.

### DNA probes

Probes representing 14 oncogenes were utilised (Table II). In addition, we used genomic probe pH 3–4 (Lalande et al., 1986) and cDNA probe p2R3.8 (Friend et al., 1986) from the retinoblastoma gene. Probe pH 3–8 was purchased from ATCC/S. Latt and p2R3.8 was provided by M. Nordenskiöld. The samples of patients 11 and 12 with malignant lymphoma were also studied with an immunoglobulin heavy joining region gene probe ($J_H$; Ravetch et al., 1981) and an immunoglobulin kappa constant region gene probe ($C_K$; Hieter et al., 1982) to confirm the histologic diagnosis. Both immunoglobulin gene probes were provided by P. Leder.

In Southern hybridisations the immunoglobulin kappa constant region gene probe from the short arm of chromosome 2 (see above) was used as a quantitative reference to detect an altered dosage. Signals obtained with probes pASφc7 (derived from the argininosuccinate synthetase pseudogene 1, Daiger et al., 1984), pNJ-3/3.5 (derived from the collagen type I alpha 2 gene locus, Tsipouras et al., 1984), pPRP112.2RP (derived from the proline-rich protein subfamily 1, Azen et al., 1984), and universal bcr (representing the breakpoint cluster region, Groffen et al., 1984) were used to represent 7p, 7q, 12p and 22q dosages, respectively. The pASφc7 probe was provided by A. Beaudet and W. O'Brien, pNJ-3/3.5 by P. Tsipouras, and universal bcr by J. Groffen. Probe pPRP112.2RP was obtained from ATCC/O. Smithies.

In RNA studies cDNA probe pHcGAP (derived from the glyceraldehyde-3-phosphate dehydrogenase gene, Tso et al., 1985) was used as a quantitative standard. Expression levels were also standardised with genomic probe pHRL83-IVS4 (Gunning et al., 1984). That represents the human cardiac actin gene locus but cross-hybridises with human β-actin mRNA. Probes pHcGAP and pHRL83-IVS4 were obtained from ATCC (depositors R. Wu and L. Kedes, respectively).

### Results

### DNA studies

Using probes listed in Table II no restriction fragments of unexpected size were observed by Southern hybridisation in

**Table II** Oncogene probes used

| Locus symbol[a] | Probe | Type of insert[b] | Chromosomal location | Provided by | Reference |
|---|---|---|---|---|---|
| NRAS | p52c- | G | 1p13 | ATCC/R. Weinberg | Murray et al., 1983 |
| MYCN | pNB-1 | G | 2p24 | ATCC/J. Bishop | Schwab et al., 1983 |
| CSF1R | pcfms104 | c | 5q33-q35 | ATCC/A. Ullrich | Coussens et al., 1986 |
| MYB | pHM2.6 | G | 6q22-q23 | ATCC/D. Stehelin | Dozier et al., 1986 |
| EGFR | pE7 | c | 7p13-p12 | ATCC/G. Merlino, I. Pastan | Xu et al., 1984 |
| PDGFA | D1 | c | 7p22-p21 or q11.2-q21.1 | C. Betsholtz | Betsholtz et al., 1986 |
| MET | MetH | G | 7q31 | G. Vande Woude | Zengerling et al., 1987 Park et al., 1988 |
| MYC | pHSR-1 | G | 8q24 | ATCC/J. Bishop | Alitalo et al., 1983 |
| ABL | pablK2 | G | 9q34 | ATCC/R. White | Srinivasan et al., 1981 |
| HRAS1 | pT24-C3 | G | 11p15.5 | ATCC/M. Barbacid | Santos et al., 1982 |
| KRAS2 | p640 | G | 12p12.1 | R. Weinberg | McCoy et al., 1983 |
| | pSW11-1 | c | | ATCC/R. Weinberg | McCoy et al., 1984 |
| | c-K-ras (Pr-1) | c | | Oncogene Science | Shimizu et al., 1983 |
| TP53 | pHp53B | c | 17p13.1 | ATCC/D. Givol | Zakut-Houri et al., 1985 |
| ERBB2 | pCER204 | c | 17q11-q12 | ATCC/T. Yamamoto | Semba et al., 1985 |
| PDGFB | pSM-1 | c | 22q12.3-q13.1 | ATCC/R. Gallo | Clarke et al., 1984 |

[a]Human Gene Mapping 10, 1989; [b]G = genomic, c = cDNA.

any tumour sample. However, the lymphoma tumours of patients 11 and 12 were characterised by clonal rearrangements of the immunoglobulin heavy chain genes (both patients) and the kappa light chain gene (patient 11) (not shown). Consistent dosage changes were found neither with oncogene probes representing the NRAS, MYCN, CSF1R, MYB, MYC, ABL, HRAS1, TP53, and ERBB2 loci, nor with probe pH 3–8. On the contrary, probes consisting of KRAS2, PDGFA, EGFR, MET, and PDGFB sequences revealed dosage alterations relative to the dosage of 2p (a reference) in tumour DNA of totally eight patients when compared to normal DNA of the same patients. In Tera-1 cell line the dosage of different oncogenes was determined using DNA from normal tissue samples of patient 16 (run in the same electrophoretic gels) for comparison (as no normal tissue of the patient from whom the Tera-1 cell line was derived was available).

*KRAS2*

In comparison with normal DNA tumour DNA of six patients with germ cell neoplasm showed an increase (1.6–3.6-fold) in KRAS2 dosage (from 12p) with respect to the reference locus $C_K$ (from 2p) (Figure 1 and Table III). There was an essentially parallel increase in the pPRP112.2RP signal (used to standardise the 12p dosage). In the Tera-1 cell line the dosage of KRAS2 was 5.2 and 1.0 relative to the immunoglobulin kappa constant region gene locus and the salivary proline-rich protein gene locus, respectively. The former ratio is in agreement with that reported previously relative to MOS (Wang *et al.*, 1987). The latter ratio suggests that the increase in the KRAS2 dosage most likely resulted from chromosomal changes involving the short arm of chromosome 12 as in the fresh tumours described above.

*PDGFA and EGFR*

Tumour DNA of five patients with germ cell neoplasm (Nos. 2, 5, 7, 13, and 14) revealed a relative increase in PDGFA dosage (Figure 2 and Table III). The alteration was evident with both $C_K$ (from 2p) and pASφc7 (from 7p) used as references. The relative intensity ratios varied between 1.8 and 3.0. The PDGFA change was accompanied by an increase in EGFR dosage in patient 7 and a borderline increase in patients 13 and 14. On the contrary, no change in EGFR was observed in patients 2 and 5 (but they showed an increase in PDGFB dosage in their tumour DNA, see below). The Tera-1 cell line showed a clearly increased intensity ratio (2.5) of EGFR relative to the 7p reference signal. Patient 12 with testicular lymphoma revealed increased dosages of PDGFA and EGFR (Figure 2 and Table III).

**Figure 1** DNA (5μg) from blood (B), normal testicular tissue (N), epididymis (E), and tumour (T) tissue of seven patients with germ cell neoplasm and DNA (T*: 2.5 μg, T: 5 μg) from the Tera-1 cell line. Tumour DNA of patients 1, 2, 5, 7, 14 and 17 showed an increase in the relative dosage of KRAS2 while patient 16 exemplifies a tumour without change. TaqI-digested DNA samples of the patients and the cell line were hybridised with p640 (representing KRAS2) and rehybridised successively with $C_K$ (a non-12p-reference) and pPRP112.2RP (a 12p-reference). p640 detects a TaqI RFLP with allelic fragments of 3.3 and 5.7 kb. Probe $C_K$ hybridises to a fragment of approximately 12 kb and pPRP112.2RP to fragments between 7 and 1.2 kb. DNA samples from patients 16, 17 and the Tera-1 cell line were electrophoresed separately from those of the others.

**Table III** Changes in the dosage of KRAS2, PDGFA, EGFR, MET, and PDGFB in tumour DNA relative to normal DNA from eight patients with testicular neoplasm and in the Tera-1 cell line

| *Patient No.* | *KRAS2/ $C_K$* | *KRAS2/ pPRP112.2RP* | *PDGFA/ $C_K$* | *PDGFA/ pASφc7* | *EGFR/ $C_K$* | *EGFR/ pASφc7* | *MET/ $C_K$* | *MET/ pNJ-3/3.5* | *PDGFB/ $C_K$* | *PDGFB/ Univ. bcr* |
|---|---|---|---|---|---|---|---|---|---|---|
| 1 | 3.6 | 1.5 | N | N | N | N | 1.8 | 1.3 | N | N |
| 2 | 1.6 | 1.0 | 2.5 | 3.3 | N | N | 3.3 | 0.8 | 2.2 | 2.3 |
| 5 | 3.5 | 1.6 | 3.0 | 2.8 | N | N | N | N | 3.0 | 3.0 |
| 7 | 1.8 | 1.0 | 2.9 | 3.3 | 2.5 | 3.3 | N | N | N | – |
| 13 | N | N | 2.7 | 1.9 | N (1.4) | 1.0 | N | N | N | N |
| 14 | 2.0 | 1.0 | 1.8 | 2.3 | N (1.4) | 1.6 | N | N | N | N |
| 17 | 1.6 | 0.8 | N | N | N | N | – | – | N | N |
| 12 | N | – | 1.7 | 1.8 | 1.7 | 1.5 | – | – | 4.0 | 2.9 |
| Tera-1 | 5.2 | 1.0 | 1.9 | 1.1 | 5.2 | 2.5 | – | – | N | N |

Abbreviations: N, no change, –, Not determined. The dosage changes are expressed as relative intensity ratios that were determined by densitometric analysis as described in the text (normal ratio = $1.0 \pm 0.4$). The dosage of each oncogene was calculated relative to the immunoglobulin kappa constant region gene locus (from 2p) and a locus from the same chromosome arm on which the oncogene is located. Densitometry was performed on several autoradiograms per oncogene whenever possible and the mean of the intensity ratios was calculated.

**Figure 2** DNA (5 μg) from blood (B), epididymis (E), and tumour (T) of eight patients with testicular malignancy and DNA (5 μg) from the Tera-1 cell line. Tumour DNA samples of patients 2, 5, 7, 12, 13, 14, and Tera-1 revealed dosage changes of PDGFA and/or EGFR whereas patients 1 and 16 are examples of cases without changes. The samples were digested with HindIII. In **a** the membrane was hybridised with a probe representing PDGFA and rehybridised with $C_K$ (a reference from 2p). The PDGFA probe and the $C_K$ probe hybridise to fragments of about 18 kb and 11 kb, respectively. In **b** the membrane was consecutively hybridised with probes for PDGFA, EGFR, and probe pASφc7 (a 7p reference). The probes detect fragments of approximately 18 kb, 17 kb and 1 kb, respectively.

### *MET*

Hybridisation with pmetH probe yielded enhanced signals in tumour DNA samples of patients 1 and 2. This probably reflected increased 7q dosages in general and was not indicative of specific amplification (Figure 3 and Table III).

**Figure 3** Blood (B) and tumour (T) DNA samples of patients 1 and 2, digested with EcoRI and hybridised and rehybridised with probes pmetH (representing MET), $C_K$ (a non-7q reference), and pNJ-3/3.5 (a 7q reference). The pmetH probe detects a fragment of about 2 kb and the $C_K$ probe a fragment of 2.5 kb. Probe pNJ-3/3.5 hybridises to allelic fragments of 13 and 3.5 kb.

### *PDGFB*

An increase in the relative dosage of PDGFB (from 22q) appeared in patients 2 and 5 with germ cell neoplasm (Figure 4 and Table III). Intensities of restriction fragments representing the universal bcr probe were comparable to those obtained with the $C_K$ probe; thus 22q polysomy was not obviously an explanation of the observed PDGFB changes. The increases in PDGFB dosage were of the same magnitude as the PDGFA changes in those two patients. Patient 12 with malignant lymphoma also showed an enhancement of PDGFB signal.

### *RNA studies*

Dot blots containing total RNA from tumour and whenever possible, normal testicular tissue of the patients were successively hybridised with probes KRAS2, PDGFA, EGFR and MYC and thereafter with probes pHcGAP and pHRL83-IVS4 (see Materials and methods) to standardise expression

**Figure 4** Normal (B, blood, E, epididymis) and tumour (T) DNA samples of patients 2, 5, and 12 showing increases in PDGFB dosage as well as the Tera-1 cell line and patient 16 representing negative findings. The samples were digested with HindIII. Hybridisation was with a probe derived from the PDGFB locus and rehybridisations were with $C_K$ (a non-22q reference) and a universal bcr probe (a 22q reference). The PDGFB probe detects a restriction fragment length polymorphism with allelic fragments of 22.6 and 19.4 kb. $C_K$ hybridises to an 11 kb fragment and the universal bcr probe to fragments of 11 kb and 4.5 kb. The samples of patients 2 and 5 were electrophoresed separately from those of the others.

levels. KRAS2, PDGFA and MYC showed detectable expression in tumour and normal testicular tissues under stringent filter washing conditions. Under similar conditions no specific signal was obtained in hybridisation of the dot blot with a probe derived from the EGFR gene (not shown). As a common observation the mRNA level of a particular oncogene varied between tumour samples as well as normal tissue samples of different individuals relative to the standards used. Of note, when the tumour of each patient was compared to normal testis from the same individual roughly equal expression levels were found. Representative examples are given in Figures 5 and 6.

Figure 5 shows serial dilutions of total RNA from 10 tumours representing different histologies as well as three normal testes from control males (see Materials and methods). The membrane was hybridised successively with probes representing the KRAS2 locus and the PDGFA locus and the hybridisation signals were compared to the $\beta$-actin mRNA levels. The relative expression of KRAS2 was clearly higher in tumours of patients 1, 5, 7, 9, 13, 14 and 15 than in tumours of patients 2, 6, and 8. On the contrary, PDGFA revealed only minor variation in expression between different samples. At rehybridisation of the blot with probe pHcGAP from the glyceraldehyde-3-phosphate dehydrogenase gene signal intensities directly proportional to those of the actin probe were obtained except in patients 1 and 5 whose tumours showed clearly elevated mRNA levels (see below).

The essential similarity of expression levels in normal and tumour tissue of the same patient is exemplified by KRAS2 in Figure 6. The tumour RNA samples (from three seminomas and three nonseminomas) shown in Figure 6 represent a part of a duplicate series of that presented in Figure 5. The dot blot was hybridised with a KRAS2 probe (a) and rehybridised with probe pHcGAP from the glyceraldehyde-3-phosphate dehydrogenase gene (b) – both probes map to the short arm of chromosome 12. In the tumours of patients 1 and 5 there was an elevation in the expression of the glyceraldehyde-3-phosphate dehydrogenase gene accompanied by a smaller increase in KRAS2 expression in patient 1 and no increase in KRAS2 expression in patient 5. This obviously reflected the presence of one or several isochromosome 12p markers and correlated well with the Southern hybridisation results that showed the highest dosages of 12p in precisely these two tumours (see above). The membrane in Figures 6a and b was further hybridised with a probe derived from the retinoblastoma gene (c). It showed similar expression levels in normal and tumour samples of the patients except in patient 5 who revealed a higher retinoblastoma gene mRNA level in normal testis relative to his tumour. Figure 6d (hybridisation result obtained with the actin probe) shows that the lanes representing tumour and normal testicular tissue samples from patient 5 with expression changes of two different kinds contained roughly equal amounts of hybridisable RNA.

### Discussion

At present only a few reports of oncogene alterations in human testis cancer exist. Cytogenetically a specific chromosome marker has been found in more than 80% of testicular germ cell tumours: an isochromosome for the short arm of chromosome 12 [i(12p)] (Castedo *et al.*, 1988; Samaniego *et al.*, 1990). The i(12p) copy number has been reportd to have prognostic significance (Bosl *et al.*, 1989). It is not known, however, which of the genes carried by 12p are associated with the pathogenetic events in testicular cancer.

The KRAS2 oncogene is one obvious candidate. The gene has been shown to be affected by point mutations or amplification in a wide variety of human tumours or tumour cell lines, including pancreas (Almoguera *et al.*, 1988), colon (Vogelstein *et al.*, 1988), and lung (Taya *et al.*, 1984). In seminoma tumours Mulder *et al.* (1989) found mutations at codons 12 or 61 of the KRAS2 gene (and the NRAS gene) with a frequency of 40%. These mutations were not, however, detected in human non-seminoma cell lines with increased copy numbers of 12p (Dmitrovsky *et al.*, 1990). Published reports of KRAS2 dosage and expression in testis cancer are mainly based on cell lines. Wang *et al.* (1987) and Dmitrovsky *et al.* (1990) observed 4–6-fold increases in KRAS2 dosage in non-seminoma cell lines relative to a non-12p reference locus. However, with a reference from 12p specific amplification of KRAS2 could not be demonstrated (Dmitrovsky *et al.*, 1990). Analogous findings were reported by Mulder *et al.* (1989) in primary seminomas. On the other hand, an increased gene dosage does not necessarily result in overexpression (Wang *et al.*, 1987) and conversely, enhanced expression may occur without any evidence of amplification at the DNA level (Monnat *et al.*, 1987). Our present findings are generally consistent with the above observations and emphasise that control probes from the same chromosome should always be used.

The PDGFA and PDGFB genes encode A- and B-chains of the platelet-derived growth factor and are located on chromosome 7p and 22q, respectively. They are expressed independently of each other in human leukaemia and solid tumour cell lines as well as in certain normal cells (Betsholtz *et al.*, 1986; Alitalo *et al.*, 1987). In a study of Slamon *et al.* (1984) PDGFB transcripts were not found in any of 54 fresh human tumours, including one germ-cell tumour. EGFR in turn is shown to be amplified in human epidermoid carcinoma (Merlino *et al.*, 1984; Hollstein *et al.*, 1988). Expression of EGFR is carefully regulated in normal cells, but its expression has been detected, apart from epidermoid carcinoma cells, in cell lines derived from the female urogenital system (Xu *et al.*, 1984). In the present study detectable (up to 3-fold) increases in DNA dosage of PDGFA, PDGFB, or

**Figure 5** Dot blot containing total RNA from testicular tumours of 10 patients (Nos. between 1 and 15) and testicular tissue of three of control males without any testicular malignancy (I–III). The blot was hybridised with probe c-K-*ras* (Pr-1) representing KRAS2 **a**, a PDGFA probe **b** and the actin probe pHRL83-IVS4 **c**. After hybridisations the membrane was washed at high stringency and exposed for 3 **a**, 4 **b**, or 6 days **c**.

**Figure 6** Dot blot containing total RNA from tumour (T) and normal testicular (N) (or epididymal, E) tissue of six patients with testicular germ cell malignancy (Nos. between 1 and 17). Hybridisations were with probes pSW11-1 representing KRAS2 **a**, pHcGAP derived from the glyceraldehyde-3-phosphate dehydrogenase gene **b**, p2R3.8 derived from the retinoblastoma gene **c**, and the actin probe pHRL83-IVS4 **d**. Exposure times of the autoradiograms were 2 **a**, 3 **b**, or 6 days **c**,**d**.

EGFR were observed in neoplastic tissue from a total of six patients out of 16. The signal enhancement in these cases exceeded that resulting from apparent aneuploidies of the respective chromosome arms (7p and 22q). As the tumours of the present series did not, however, reveal enhanced expression of PDGFA or EGFR in RNA dot blot assays the significance of the above amplifications remains unknown so far.

Other oncogenes reported to have been involved in testicular neoplasia include members of the *myc* family. Sikora *et al.* (1985) observed increased expression of MYC in seminomas. These authors also reported correlation between MYC expression and the grade of differentiation of a teratoma tumour: the more undifferentiated a teratoma the lower level of detectable c-*myc* protein. An elevated expression of MYCN (in the absence of gene amplification) was detected in one seminoma tumour by Saksela *et al.* (1989). Definite alterations in the *myc* genes were not observed in the present study.

In addition to oncogenes, we studied the dosage and expression of the retinoblastoma susceptibility gene that represents tumour suppressor genes. Structural alterations and/or reduced expression of this gene have been observed, apart from retinoblastoma and osteosarcoma, in breast cancer (Lee *et al.*, 1988), small cell lung cancer (Harbour *et al.*, 1988), and also in a germ cell tumour of the testis (Saksela *et al.*, 1989). Two-fold or greater increases in dosage and expression of the retinoblastoma gene were in turn reported by Gope *et al.* (1990) in human colorectal carcinomas. Except for the finding of a lower level of expression in tumour *vs* normal tissue in a single case further evidence of the retinoblastoma gene playing a role in testis tumours did not come out in the present investigation.

There was a slight over-representation of nonseminoma and clinical stage greater than I, but no straightforward association of oncogene changes with the clinical stage or the histologic subtype was found in this tumour series. It is noteworthy that all primary tumours that produced elevated serum levels of tumour markers were also characterised by changes in oncogene dosage. Oncogene changes in the present study and allelic dosage changes reported previously (Peltomäki *et al.*, 1990), though causally related in some cases, coexisted in several tumours. The situation is analogous to colon carcinoma where multiple steps – DNA hypomethylation, loss of alleles on chromosomes 5, 7 and 18, and activation of *ras* oncogenes – ultimately lead to a malignant phenotype (Vogelstein *et al.*, 1988).

We thank the investigators who provided us with the probes used in this study. We are grateful to Dr Antero Halme and the physicians and staff of the Second and Fourth Departments of Surgery at the Helsinki University Central Hospital for help in providing the patient samples. This work was supported by the Academy of Finland, the Finnish Cancer Society, the Paulo Foundation, and the Sigrid Jusélius Foundation. This study was carried out in part at the Folkhälsan Institute of Genetics.

### References

ALITALO, K., SCHWAB, M., LIN, C.C., VARMUS, H.E. & BISHOP, J.M. (1983). Homogeneously staining chromosomal regions contain amplified copies of an abundantly expressed cellular oncogene (c-*myc*) in malignant neuroendocrine cells from a human colon carcinoma. *Proc. Natl Acad. Sci. USA*, **80**, 1707.

ALITALO, R., ANDERSSON, L.C., BETSHOLTZ, C. & 4 others (1987). Induction of platelet-derived growth factor gene expression during megakaryoblastic and monocytic differentiation of human leukemia cell lines. *EMBO J.*, **6**, 1213.

ALMOGUERA, C., SHIBATA, D., FORRESTER, K., MARTIN, J., ARNHEIM, N. & PERUCHO, M. (1988). Most human carcinomas of the exocrine pancreas contain mutant c-K-*ras* genes. *Cell*, **53**, 549.

AZEN, E., LYONS, K.M., MCGONIGAL, T. & 6 others (1984). Clones from the human gene complex coding for salivary proline-rich proteins. *Proc. Natl Acad. Sci. USA*, **81**, 5561.

BETSHOLTZ, C., JOHNSSON, A., HELDIN, C.-H. & 9 others (1986). cDNA sequence and chromosomal localization of human platelet-derived growth factor A chain and its expression in tumour cell lines. *Nature*, **320**, 695.

BIANCHI, N.O.B., PELTOMÄKI, P., BIANCHI, M.S., KNUUTILA, S. & DE LA CHAPELLE, A. (1988). Demethylation of two specific DNA sequences in expressed human immunoglobulin light kappa constant genes. *Somatic Cell. Mol. Genet.*, **14**, 13.

BOSL, G.J., DMITROVSKY, E., REUTER, V.E. & 4 others (1989). Isochromosome of chromosome 12: clinically useful marker for male germ cell tumors. *J. Natl Cancer Inst.*, **81**, 1874.

CASTEDO, S.M.M.J., DE JONG, B., OOSTERHUIS, J.W. & 4 others (1988). i(12p)-negative testicular germ cell tumors. A different group? *Cancer Genet. Cytogenet.*, **35**, 171.

CHOMCZYNSKI, P. & SACCHI, N. (1987). Single-step method of RNA isolation by acid guanidinium thiocyanate-phenol-chloroform extraction. *Anal. Biochem.*, **162**, 156.

CLARKE, M.F., WESTIN, E., SCHMIDT, D. & 5 others (1984). Transformation of NIH3T3 cells by a human c-*sis* DNA clone. *Nature*, **308**, 464.

COUSSENS, L., BEVEREN, C.V., SMITH, D. & 5 others (1986). Structural alterations of viral homologue of receptor proto-oncogene *fms* at carboxyl terminus. *Nature*, **320**, 277.

DAIGER, S.P., HOFFMANN, N.S., WILDIN, R.S. & SU, T.-S. (1984). Multiple independent restriction site polymorphisms in human DNA detected with a cDNA probe to argininosuccinate synthetase (AS). *Am. J. Hum., Genet.*, **36**, 736.

DMITROVSKY, E., MURTY, V.V.V.S., MOY, D. & 6 others (1990). Isochromosome 12p in nonseminoma cell lines: karyologic amplification of c-Ki-*ras*2 without point mutational activation. *Oncogene*, **5**, 543.

DOZIER, C., WALBAUM, S., LEPRINCE, D. & STEHELIN, D. (1986). EcoRI RFLP linked to the human *myb* gene. *Nucleic Acids Res.*, **14**, 1928.

FEINBERG, A.P. & VOGELSTEIN, B. (1984). A technique for radiolabelling DNA restriction endonuclease fragments to high specific activity. *Anal. Biochem.*, **137**, 266.

FOGH, J. (1978). Cultivation, characterization, and identification of human tumor cells with emphasis on kidney, testis, and bladder tumors. *Natl Cancer Inst. Monogr.*, **49**, 5.

FRIEND, S.H., BERNARDS, R., ROGELJ, S. & 4 others (1986). A human DNA segment with properties of the gene that predisposes to retinoblastoma and osteosarcoma. *Nature*, **323**, 643.

GOPE, R., CHRISTENSEN, M., THORSON, A. & 4 others (1990). Molecular analysis of the retinoblastoma (Rb) gene in human colorectal carcinomas. In *Hereditary Colorectal Cancer*. Proceedings of the Fourth International Symposium on Colorectal Cancer (ISCC-4), November 9–11, 1989, Kobe, Japan. Utsunomiya, J. & Lynch, H.T. (eds). Springer-Verlag: Tokyo, p. 489.

GUNNING, P., PONTE, P., KEDES, L., HICKEY, R.J. & SKOULTCHI, A.J. (1984). Expression of human cardiac actin in mouse L cells: a sarcomeric actin associates with a nonmuscle cytoskeleton. *Cell*, **36**, 709.

HARBOUR, J.W., LAI, S.-L., WANG-PENG, J., GAZDAR, A.F., MINNA, J.D. & KAYE, F.J. (1988). Abnormalities in structure and expression of the human retinoblastoma gene in SCLC. *Science*, **241**, 353.

HIETER, P.A., MAIZEL, J.V. & LEDER, P. (1982). Evolution of human kappa J region genes. *J. Biol. Chem.*, **257**, 1516.

HOLLSTEIN, M.C., SMITS, A.M., GALIANA, C. & 5 others (1988). Amplification of epidermal growth factor receptor gene but no evidence of *ras* mutations in primary human esophageal cancers. *Cancer Res.*, **48**, 5119.

HUMAN GENE MAPPING 10 (1989). *Cytogenet. Cell Genet.*, **51**, 1.

KUNKEL, L.M., SMITH, K.D., BOYER, S.H. & 6 others (1977). Analysis of human Y-chromosome-specific reiterated DNA in chromosome variants. *Proc. Natl Acad. Sci. USA*, **74**, 1245.

LALANDE, M., DONLON, T., PETERSEN, R.A., LIBERFARB, R., MANTEL, S. & LATT, S.A. (1986). Molecular detection and differentiation of deletions in band 13q14 in human retinoblastoma. *Cancer Genet. Cytogenet.*, **23**, 151.

LEE, E.Y.-H.P., TO, H., SHAW, J.Y., BOOKSTEIN, R., SCULLY, P. & LEE, W.-H. (1988). Inactivation of the retinoblastoma susceptibility gene in human breast cancers. *Science*, **241**, 218.

LOTHE, R.A., FOSSÅ, S.D., STENWIG, A.E. & 4 others (1989). Loss of 3p or 11p alleles is associated with testicular cancer tumors. *Genomics*, **5**, 134.

MANIATIS, T., FRITSCH, E.F. & SAMBROOK, J. (1982). Isolation of high-molecular-weight, eukaryotic DNA from cells grown in tissue culture. In *Molecular Cloning*. Cold Spring Harbor Laboratory, Cold Spring Harbor: New York, p. 280.

MCCOY, M.S., BARGMANN, C.I. & WEINBERG, R.A. (1984). Human colon carcinoma. Ki-*ras*2 oncogene and its corresponding proto-oncogene. *Mol. Cell. Biol.*, **4**, 1577.

MCCOY, M.S., TOOLE, J.J., CUNNINGHAM, J.M., CHANG, E.H., LOWY, D.R. & WEINBERG, R.A. (1983). Characterization of a human colon/lung carcinoma oncogene. *Nature*, **302**, 79.

MERLINO, G.T., XU, Y.H., ISHII, S. & 5 others (1984). Amplification and enhanced expression of the epidermal growth factor receptor gene in A431 human carcinoma cells. *Science*, **244**, 417.

MONNAT, M., TARDY, S., SARAGA, P., DIGGELMANN, H. & COSTA, J. (1987). Prognostic implications of expression of the cellular genes *myc*, *fos*, Ha-*ras* and Ki-*ras* in colon carcinoma. *Int. J. Cancer*, **40**, 293.

MOSTOFI, F.K. & SOBIN, L.K. (1977). Histological typing of testis tumors. In *International Histological Classification of Tumours*. No. 16. World Health Organization: Geneva.

MULDER, M.P., KEIJZER, W., VERKERK, A. & 4 others (1989). Activated *ras* genes in human seminoma: evidence for tumor heterogeneity. *Oncogene*, **4**, 1345.

MURRAY, M.J., CUNNINGHAM, J.M., PARADA, L.F., DAUTRY, F., LEBOWITZ, P. & WEINBERG, R.A. (1983). The HL-60 transforming sequence: a *ras* oncogene coexisting with altered *myc* genes in hematopoietic tumors. *Cell*, **33**, 747.

PARK, M., TESTA, J.R., BLAIR, D.G., PARSA, N.Z. & VANDE WOUDE, G.F. (1988). Two rearranged MET alleles in MNNG-HoS cells reveal the orientation of MET on chromosome 7 to other markers tightly linked to the cystic fibrosis locus. *Proc. Natl Acad. Sci. USA*, **85**, 2667.

PECKHAM, M. (1988). Testicular cancer. *Rev. Oncol.*, **1**, 439.

PELTOMÄKI, P., HALME, A. & DE LA CHAPELLE, A. (1989). Molecular studies of the sex chromosomes in human testicular cancer: pronounced changes in X and Y chromosome dosage in some tumors. *Genes, Chromosomes Cancer*, **1**, 42.

PELTOMÄKI, P., HALME, A. & DE LA CHAPELLE, A. (1990). Human testicular cancer: changes in autosomal dosage. *Cancer Genet. Cytogenet.*, **48**, 1.

PUGH, R.C.B. (1976). Testicular tumours. Introduction. In *Pathology of the Testis*. Blackwell: Oxford, p. 139.

RADICE, P., PIEROTTI, M.A., LACERENZA, S. & 4 others (1989). Loss of heterozygosity in human germinal tumors. *Cytogenet. Cell. Genet.*, **52**, 72.

RAVETCH, J.V., SIEBENLIST, U., KORSMEYER, S.J., WALDMANN, T. & LEDER, P. (1981). Structure of the immunoglobulin $\mu$ locus: characterization of embryonic and rearranged J and D genes. *Cell*, **27**, 583.

RIGBY, P.W.J., DIECKMANN, M., RHODES, C. & BERG, P. (1977). Labelling deoxyribonucleic acid to a high specific activity *in vitro* by nick translation with DNA polymerase I. *J. Mol. Biol.*, **113**, 237.

RUKSTALIS, D.B., BUBLEY, G.J., DONAHUE, J.P., RICHIE, J.P., SEIDMAN, J.G. & DE WOLF, W.C. (1989). Regional loss of chromosome 6 in two urological malignancies. *Cancer Res.*, **49**, 5087.

SAKSELA, K., MÄKELÄ, T.P. & ALITALO, K. (1989). Oncogene activation in small cell lung cancer and a germ tumor of the testis: activation of the N-*myc* gene and decreased RB mRNA. *Int. J. Cancer*, **44**, 182.

SAMANIEGO, F., RODRIGUEZ, E., HOULDSWORTH, J. & 10 others (1990). Cytogenetic and molecular analysis of human male germ cell tumors: chromosome 12 abnormalities and gene amplification. *Genes Chromosomes Cancer*, **289**, 1990.

SANTOS, E., TRONICK, S.R., AARONSON, S.A., PULCIANI, S. & BARBACID, M. (1982). T24 human bladder carcinoma oncogene is an activated form of the normal human homologue of BALB- and Harvey-MSV transforming genes. *Nature*, **298**, 343.

SCHWAB, M., ALITALO, K., KLEMPNAUER, K.-H. & 6 others (1983). Amplified DNA with limited homology to *myc* cellular oncogene is shared by human neuroblastoma cell lines and a neuroblastoma tumor. *Nature*, **305**, 245.

SEMBA, K., KAMATA, N., TOYOSHIMA, K. & YAMAMOTO, T. (1985). A v-*erb*B-related proto-oncogene, c-*erb*B-2, is distinct from the c-*erb*B-1/epidermal growth factor-receptor gene and is amplified in a human salivary gland adenocarcinoma. *Proc. Natl Acad. Sci. USA*, **82**, 6497.

SHIMIZU, K., GOLDFARB, M. & SUARD, Y. (1983). Three human transforming genes are related to the viral *ras* oncogenes. *Proc. Natl Acad. Sci. USA*, **80**, 2112.

SIKORA, K., EVANS, G., STEWART, J. & WATSON, J.V. (1985). Detection of the c-*myc* oncogene product in testicular cancer. *Br. J. Cancer*, **52**, 171.

SLAMON, D., DEKERNION, J.B., VERMA, I.M. & CLINE, M.J. (1984). Expression of cellular oncogenes in human malignancies. *Science*, **224**, 256.

SOUTHERN, E.M. (1975). Detection of specific sequences among DNA fragments separated by gel electrophoresis. *J. Mol. Biol.*, **98**, 503.

SRINIVASAN, A., REDDY, E.P. & AARONSON, S.A. (1981). Abelson murine leukemia virus: molecular cloning of infectious integrated proviral DNA. *Proc. Natl Acad. Sci. USA*, **78**, 2077.

TAYA, Y., HOSOGAI, K., HIROHASHI, S. & 6 others (1984). A novel combination of K-*ras* and *myc* amplification accompanied by point mutational activation of K-*ras* in human lung cancer. *EMBO J.*, **3**, 2943.

TSIPOURAS, P., BØRRESEN, A.-L., DICKSON, L.A., BERG, K., PROCKOP, D.J. & RAMIREZ, F. (1984). Molecular heterogeneity in the mild autosomal dominant forms of osteogenesis imperfecta. *Am. J. Hum. Genet.*, **36**, 1172.

TSO, J.Y., SUN, X.-H., KAO, T., REECE, K.S. & WU, R. (1985). Isolation and characterization of rat and human glyceraldehyde-3-phosphate dehydrogenase cDNAs: genomic complexity and molecular evolution of the gene. *Nucleic Acids Res.*, **13**, 2485.

WANG, L., VASS, W., GAO, C. & CHANG, K.S.S. (1987). Amplification and enhanced expression of the c-Ki-*ras*2 proto-oncogene in human embryonal carcinomas. *Cancer Res.*, **47**, 4192.

VOGELSTEIN, B., FEARON, E.R., HAMILTON, S.R. & 7 others (1988). Genetic alterations during colorectal-tumor development. *N. Engl. J. Med.*, **319**, 525.

XU, Y., RICHERT, N., ITO, S., MERLINO, G.T. & PASTAN, I. (1984). Characterization of epidermal growth factor receptor gene expression in malignant and normal human cell lines. *Proc. Natl Acad. Sci. USA*, **81**, 7308.

ZAKUT-HOURI, R., BIENZ-TADMOR, B., GIVOL, D. & OREN, M. (1985). Human p53 cellular tumor antigen: cDNA sequence and expression in COS cells. *EMBO J.*, **4**, 1251.

ZENGERLING, S., OLEK, K., TSUI, L.-C., GRZESCHIK, K.-H., RIORDAN, J.R. & BUCHWALD, M. (1987). Mapping of DNA markers linked to the cystic fibrosis locus on the long arm of chromosome 7. *Am. J. Hum. Genet.*, **40**, 228.